# Attributes development for a discrete choice experiment on preferences in sexual and reproductive health services for adolescents and young people in Nigeria

**DOI:** 10.1186/s12913-022-08888-1

**Published:** 2022-12-12

**Authors:** Olujide Arije, Jason Madan, Tintswalo Hlungwani

**Affiliations:** 1 Institute of Public Health, Obafemi Awolowo University, Ile-Ife, Nigeria; 2 School of Public Health, University of the Witwatersrand, Johannesburg, South Africa; 3 Warwick Medical School, University of Warwick, Warwick, UK

**Keywords:** Stated preferences, Discrete choice experiment, Attribute-level development, Mixed methods, Modified Delphi, Nigeria

## Abstract

**Background:**

A major component of the validity of the discrete choice experiment (DCE) research design lies in the correct specification of attributes and levels relevant to the research focus. In this paper, we set out the validation steps we took in designing the tool for a DCE on preferences in sexual and reproductive health (SRH) services for adolescents and young people.

**Methodology:**

This study was carried out among adolescents and young people (AYP) in Ogun State, Southwest Nigeria. We used a three-step mixed-methods process in developing the attributes and attribute-levels for our DCE tool. The first was to conduct a series of 16 focus group discussions (FGD) with AYP ensuring maximal variation (by age group, sex, marital status, and location). The FGD included a priority listing process in which participants were asked to list and rank the most important characteristics of optimal SRH services for AYP. The lists were harmonized and items were scored. The main (highest scoring) themes emerging from the harmonized priority list were converted into an initial set of attributes and the subthemes as level. These initial attributes and levels were presented to a panel of methods and content experts in a virtual modified Delphi process. This was for deciding on the importance of the attributes in providing optimum sexual and reproductive health services for young people, and the appropriateness of the levels. The same set of attributes was presented to another set of AYP in a series of four FGD to clarify meanings, and test whether the wordings were well understood. We applied some decision rules for including and excluding attributes and levels in the different phases of the development process.

**Results:**

We extracted an initial set of nine attributes with 2-4 levels each from the first FGD sessions. These were revised to a final set of seven attributes with 2-4 levels each based on findings from the expert review and final validation FGDs with AYP. The final attributes were: the type of staff, physical environment, health worker attitude, cost, waiting time, contraceptive availability, and opening hours.

**Conclusion:**

The final set of attributes covered those relating to the services provided, the health workers providing the services, and the AYP. Our three-step process which included both quantitative and qualitative approaches ensured a rigorous process that produced a reliable combination of attributes and levels. Although we had to trade off some competing attributes to come to a final list, our decision rules helped us to conduct a transparent and reproducible process.

## Introduction

Adolescents and young people (AYP) constitute over one-quarter of the global population, and about 90% live in low and middle-income countries [1]. The health of AYP is taking center stage in health services in many developing countries, with a focus on programs that reduce adverse health outcomes among this population [2]. The focus on adolescents and young people by the WHO and partners is seen in their *Global Accelerated Action for the Health of Adolescents* (AA-HA!) as well as the *Global Standards for quality healthcare services for adolescents* program, which are designed to guide implementation of adolescent health-related services at national levels [3]. However, adolescent and youth-friendly services are still widely unavailable [4–6], with most of the services provided at the public care facilities focused on care for the general population [7, 8]. Meanwhile, there are significant sexual and reproductive health (SRH) challenges among adolescents and young people [9].

Typically, adolescent health services can be offered at facilities either as integrated or standalone services, in schools both as outreach and school-based services, and in the community as outreach services [6, 10]. While these different approaches have their pros and cons, facility-based services will perhaps continue to be required as a fulcrum of operation for all other types of service delivery approach. It is not always feasible to build and operate dedicated services for adolescents. It is perhaps easier and of more necessity to make existing services more adolescent and young people friendly [12]. Attempts to expand the current facility-based services to accommodate adolescent health services require the understanding of the important attributes of adolescent and youth friendly health services. One of such is that AYP should be involved in the design, implementation and evaluation of services targeting them [11]. As would be expected, personal preferences and perception about quality of care play critical role in the utilization of health services generally, and even more specifically for AYP.

Discrete choice models have become the tool of choice to understand consumer preferences and behavior, with essential applications in healthcare services [13]. A very important component of the validity of the discrete choice experiments (DCE) design lies in correct specification of relevant attributes and levels for the research tool. Correct specification is ensured by incorporation of a detailed and contextual understanding of the target population’s point of view [14]. The most recent guidelines for conducting DCE stipulate the necessity to describe and show the process of development of attributes and levels of a planned experiment as a validity check [15]. These guidelines were developed on the backdrop of the brevity of description of the development of the attributes and levels in several DCE studies [15]. The guidelines also recommend that qualitative methods should be used to synthesize attributes and levels of intended choice experiments. For instance, a thematic content analysis of transcripts from focus group discussions (FGD) and or key informant interviews (KII) with members of the target population was suggested [15–18].

In addition, part of what is required to be reported include the rationale for the method used to develop attributes for the DCE; study population for development process and sampling procedures used; who conducted the interviews and in what setting; data handling and analysis for the data collected; description of the results, including information about how many potential attributes were generated, and how they were manipulated to become the final set of attributes; and whether any attribute might prove problematic in the DCE. Kløjgaard et al. demonstrated the benefit of an exploratory stepwise qualitative approach starting with literature review, interaction with key stakeholders, and then interaction with potential respondents of the DCE tool [19]. Using these multiple steps, they were able to clarify what attributes and levels to include by checking that the ones developed were meaningful and able to capture all the relevant issues. They also suggested clarifying the meaning of the attributes and levels by checking that the words used were easily understandable. The objective of this study is to demonstrate the validation steps we took in designing the tool for our discrete choice experiment on preferences in sexual and reproductive health services for adolescents and young people.

## Methods

### Study setting

In this study, we report the development of a preference-elicitation tool. The tool is intended for identifying attributes of SRH services that AYP value and how strongly they value these attributes relative to each other in public health facilities. There is a draught of research in Nigerian settings that explores stated preferences in SRH of AYP. This study also comes at a time where there is increase in the interest of researchers and policy makers in the sexual and reproductive health of adolescents [4, 20, 21]. This is more so in the study location, Ogun State Nigeria, where the state government launched the *Ogun State Adolescents and young people’s sexual and reproductive health strategic framework, 2018 – 2022* in 2019 [22]. Our research stands at a policy junction in supporting the evaluation of the impact of the policy, and exploring the gaps and challenges that may be present in implementing adolescent and young people’s SRH programs.

### Study design

This study had a mixed methods design using both qualitative and quantitative methods. We used a step-wise process that included a series of FGDs with adolescents and young people for extracting potential attributes and their corresponding levels; a review of the appropriateness and importance of the proposed attributes and levels by a panel of content experts; and another series of FGDs with AYP to further clarify the meaning of the attributes and the levels, including the wordings used to present them.

### Study location

The initial and final sets of FGDs were carried out among AYP in two selected local government areas (LGA) of Ogun State, Southwest Nigeria, namely, Abeokuta South LGA (predominantly urban), and Ijebu East LGA, (predominantly rural). The expert validation was carried out virtually, and participants were from state, national, and international levels.

### Initial focus group discussions

We conducted a series of 16 focus group discussions (FGD) among AYP across the study locations. The objective of the FGD was to assess the perceptions and preferences of AYP in SRH in public health facilities in the study location. Participants were purposively selected from communities within the two study LGAs ensuring maximum variation by age group (15-19/20-24 years), sex (male/female), marital status (married/unmarried) and location (rural/urban). All FGD sessions were homogenous for each of these four characteristics. An FGD guide that explored perceptions and preferences in SRH services among AYP was used by appropriately trained moderators and note-takers in each discussion session. Each of the sessions was audio-recorded after seeking permission from the participants. The sessions were conducted in English and Yoruba languages which are the predominant languages in the study area. Towards the end of each session, participants were asked to individually list five aspects of the quality of sexual and reproductive health services of the highest priority to them, and rank these in order of importance on sheets of paper given to them.

We extracted themes and subthemes from the FGD data using thematic content analysis [23]. Only the major themes and subthemes relating to preferences of AYP in SRH are reported in this study, alongside with the analysis of the priority listing. We also present some quotations from some of the discussions sessions as evidence for the attributes we developed. The full findings from the FGD sessions are presented elsewhere [24]. The participants’ priority lists were harmonized and standardized for wording. Each unique item retained the ranking assigned to the original item on the priority list of each of the FGD participants, from 1^st^ to 5^th^ positions. These were weighted by reversed scoring from 5 to 1. A total weighted sum score for each item was obtained by the summation of the reversed scores of each unique item. The final ranking of the priority list items, based on the frequencies of the unique items as well as their weighted sum scores, was used to support identifying the essential attributes of the priority/preferred sexual and reproductive health services from the participants' perspectives.

The highest rated items emerging from the harmonized priority list were converted into an initial set of attributes with two to four levels each using three decisions rules. First, the attribute had to occur as a theme in the qualitative analysis of the discussion sessions as well as a unique category on the priority list. Secondly, the frequency of mentions of the theme in the qualitative analysis of the discussion sessions and the weighted sum score of the priority category must be relatively high, however, we did not set a cut-off point for either. The third rule was the use of expert and practical knowledge of implementation of SRH services.

### Expert Validation

Twenty adolescent sexual and reproductive health (ASRH) content and methods experts were invited to complete an online questionnaire in the expert validation step. We identified these experts through personal communication and snowballing, as well as from their scientific publications. Efforts were made to include experts from the State and National Ministries of Health, international non-governmental organization (iNGO) functioning in the study state as well as memebrs of the academia. In all, 15 people completed the questionnaire.

The initial set of attributes and corresponding levels were presented to the experts to grade in a virtual modified Delphi process. The Delphi technique is a method devised to identify the collective opinion of experts [25]. The technique typically involves contribution of opinions of a panel of experts on a matter of interest (usually in a listing format), priority scoring of the listed items by the experts, statistical aggregation of the scores, and multiple iteration of scoring with controlled feedback of group opinion. When one or more of these steps are not followed, it can be considered a modified Delphi. In this study, each participant received an email inviting them to participate, including instructions and the link to the online questionnaire. The participants were required to rate each attribute on its importance, and each level of the attributes on their appropriateness, as design features of SRH services for AYP. Importance was defined as the significance of the attribute as a component of optimal sexual and reproductive health services for young people, and appropriateness was defined as the correctness of choosing the option as one of the plausible characteristics of the corresponding design feature in the research setting. Attributes were rated from important to unimportant, while levels were rated from appropriate to inappropriate, both on a five-point rating scale. The participants also had the opportunity to make comments on each attribute and level, suggest additional levels for each attribute, and suggest new attributes for our consideration.

The inter-quartile range (IQR) of the scores for each question item was calculated. For each question item, the lower the IQR, the higher consensus amongst the reviewers. This means that the items with lower IQR were the more useful items for achieving the aim of the study. While there is no standard value, we considered an IQR of 1.0 or less adequate. Only one round of expert validation was conducted since there was no need for a first round in which experts would typically suggest items for considerations [26]. Also, there was high agreement among the participants after the first round, so further rounds were not considered necessary.

### Validation with AYP

Following the expert validation step, the same set of attributes and levels were presented to AYP in a series of four FGD sessions to assess clarity of the attributes and levels to potential users, as well as to test their comprehension of the wordings used. The study participants were AYP aged 15 – 24 years who were purposively selected from the two study LGA. They were recruited to participate by community mobilizers who were familiar with the communities. We ensured homogeneity and variation of participant by sex (male/female), and location (rural/urban) during the FGD sessions.

The FGD sessions were moderated by OA and supported by a note taker. The FGD guide we used explored characteristics of a good public health facilities for addressing the sexual and reproductive health challenges of AYP. It also explored each attribute and its corresponding levels to ask if they felt they were important as features of an SRH service for AYP, and why. The moderator read out the attributes and the levels one by one asking for the participants’ understanding of the items, as well as, whether they represented the feature they would expect in an SRH service for AYP. Finally, each participant in each session was asked to rank the items in the order of importance to them. In the last two of the sessions, group ranking was introduced alongside individual ranking.

As in the initial FGDs, the final FGD sessions were conducted in English and Yoruba Languages. They were audio-recorded, and the recordings were transcribed verbatim afterwards. Portions conducted in Yoruba Language were transcribed into English Language. The transcripts were coded in ATLAS.ti 22 using a thematic content approach. The themes explored were the attributes being validated. Subthemes were inductively created to highlight the key discussions around each of the attributes.

For the ranking of the attributes, the mean for each attribute was calculated as the arithmetic mean of the ranks assigned to them by the participants. A simple ranking of the means of the attribute ranks from the lowest to the highest represented the ranked preference for the attributes as an SRH service design feature, from most to least important, at both individual and group levels.

## Results

### Initial FGD with AYP

**Fig. 1:**
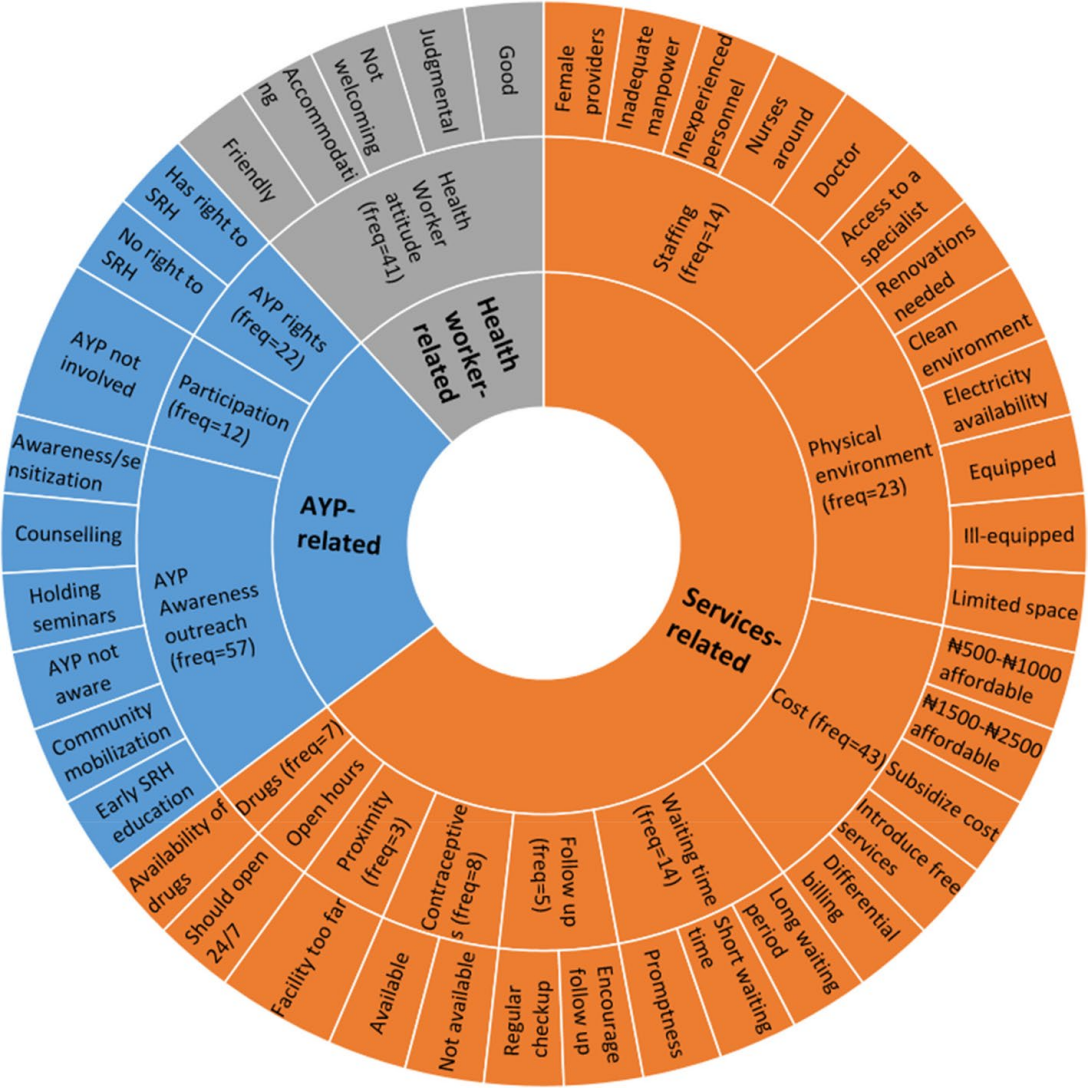
Themes and subthemes related to preferences of AYP in SRH services extracted from FGD. AYP: Adolescent and young people; FGD: Focus group discussion; Freq: Frequency; SRH: Sexual and reproductive health

While the full account of the qualitative findings of the initial FGDs with AYP is described elsewhere [24], here, we present a summary of the findings relevant to our synthesis of the preferences of the participants in SRH services in public health facilities. Figure 1 shows the themes and subthemes, extracted from FGD analysis, that are relevant to service preferences. The themes are grouped into services-related, health worker-related and AYP-related. The themes include cost of the services, health worker attitudes, physical environment, and type of staff in attendance, provision of privacy/confidentiality among others. The emerging subthemes for each theme demonstrated either negative or positive perceptions, as well as preferences and expectations of the participants with respect to SRH services. The frequencies of the themes give a sense of how grounded they were in the FGD participants’ comments and contributions.

**Fig. 2:**
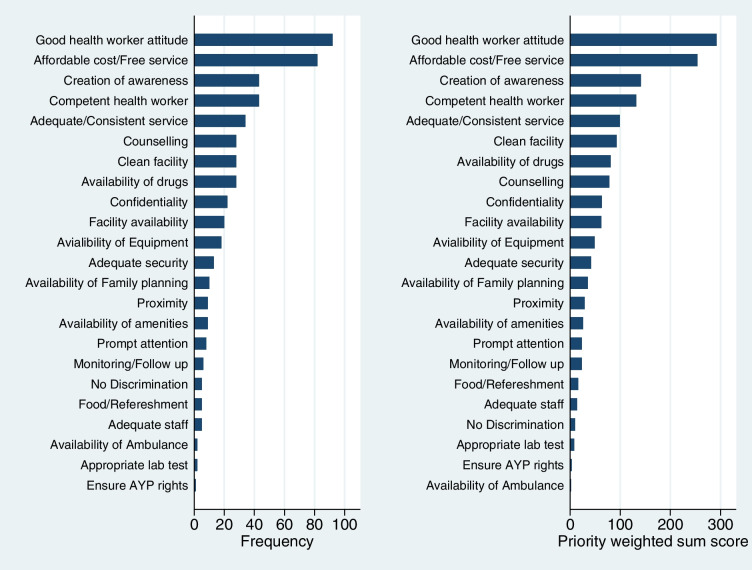
Frequency distribution and weighted sum scores of listed priority areas AYP: Adolescent and young people

For the priority list, there were 515 priority items listed across all participants, and these were reduced to 23 categories. Figure 2 shows the frequencies distribution of the reduced categories as well as the weighted sum scores for each category based on the ranking given by the FGD participants. This chart indicates that generally, the items that occurred most frequently were also rated higher than the others by the participants.

Although, *‘Proximity of health facility’*, *‘Health workers carrying out follow-up’*, and ‘*AYP rights and participation*’ were present both among the themes and in the priority lists, their weighted sum scores were low so they were not included in the attribute lists. The theme ‘*Drug availability’* seem to have been mentioned mostly in the context of drugs for managing commonly endemic illness and not specifically for drugs related to SRH. Therefore, we decided to exclude it as an attribute. We retained waiting time even though it had a relatively low weighted sum score and opening hours, although it did not have a corresponding category in the priority list. We did this because quality of care studies in adolescent health generally views them as factors that affect the utilization of services by AYP [27–29]. In Table 1 we present excerpts from discussion sessions to support the nine attributes that were selected.

**Table 1:** Attribute and supporting quotes excerpted from transcribed discussion sessions

Attribute	*Quotes excerpted from transcribed discussion sessions*
Type of staff	*“…they prefer to go to the doctor so that he will prescribe a good drug from them.***Rural/Married/Male/15-19 years**
Physical environ/Amenities	*P7: That’s what am talking about since they are health worker, they will make sure that their environment is clean. And since they want to counsel people, they will not want to do it in a way that the noise will be much that that they will not want to let people know what they are saying.***Urban/Unmarried/Female/20 - 24 years**
HW attitude	*“…I will like to emphasize on what you said, I want to expatiate. You know there is this African mentality when a youth goes into the hospital to complain about sexual health challenges he is facing, those attending to them will just feel like “ah, ah” you are doing this shit... So, they stigmatize, I will use the word stigmatization***Urban/Unmarried/Male/20 - 24 years**
AYP Outreach/Awareness creation	*“…That is my view of going from house to house and giving orientation, then parents in a way will be in support while some parents will not, that’s where orientation should come up first, because parents that don’t know about it will not encourage them.***Urban/Married/Female/20 - 24 years**
Cost	*“…In my own opinion, it is about reduction in payment so that people can be able to afford those particular services.***Urban/Unmarried/Male/20 - 24 years**
Privacy/Confidentiality	*“…most of them [health workers] they do not know how to keep secret.”***Rural/Unmarried/Male/20 - 24 years**
Waiting time	*“…they prefer to go to the chemist because they will be attended to on time than if the go to the hospital***Urban/Married/Male/20 - 24 years**
Contraceptive availability	*“They should make services available anytime they need it”.***Rural/Unmarried/Male/20 - 24 years**
Open hours	*… I don’t think there should be a limit to the time of closing hour or opening hour because there should be a time shifting here within the staff and because there is no time the patient cannot come in for treatment so that thing is causing most issues at times when we get to general hospitals and any hospitals at some hours, they will say they can’t yes and I don’t think that should be a part of.***Urban/Unmarried/Male/15-19 years**

### Expert validation

The expert validation questionnaire was completed by 15 out of the 20 people invited to participate. Their age range was 35 – 61 years. Of these, 10 were females, 8 had at least a master’s degree while two also had a PhD. The number of years of involvement in ASRH work among the participant was 9 – 30 years. All the participants carried out their work in ASRH in Nigeria apart from one person who reported the country of practice as global.

Table 2 shows the interquartile ranges for all the attributes and levels. Among the attributes, none had an IQR of more than 1 indicating a high level of agreement about their importance as a design feature for SRH for AYP. For attribute *‘Preferred health worker’*, the level ‘*Other health worker*’ had an IQR of 2; for attribute ‘*AYP Outreach/Awareness creation’*, the level ‘*Only health facility based’* had an IQR of 2; for attribute ‘*Cost of services’*, the level *‘₦1500’* had an IQR of 2; for attribute ‘*Privacy/Confidentiality’*, the level ‘*Separate space for ASRH’* had an IQR of 2; for attribute ‘*Waiting time’*, the level ‘*60 minutes’* had an IQR of 2; for attribute ‘*Contraceptive’s availability’*, the level ‘*May not be available’* had an IQR of 3; and for attribute *‘Opening hours’*, the level ‘Week day working hours only’ had an IQR of 2. All other levels had an IQR of ≤1.

**Table 2:** Interquartile ranges of attributes and levels from expert validation

Attributes and levels	IQR
**Preferred health worker**	**1**
Nurse	1
Doctor	1
Other health worker	2
**Physical environment/Amenities**	**0**
Clean and Conducive	0
Not clean or conducive	1
**Health worker attitude**	**0**
Service provider is open and friendly	0
Service provider may be stern and is judgmental	1
**AYP Outreach/Awareness creation**	**1**
Includes outreach programs	1
Only health facility based	2
**Cost of services**	**0**
Free	1
₦500	1
₦1000	1
₦1500	2
**Privacy/Confidentiality**	**0**
Separate space for ASRH	2
No separate space for ASRH	1
**Waiting time**	**1**
No wait	1
30 minutes.	1
60 minutes.	2
**Contraceptive availability**	**0**
Always available	0
May not be available	3
**Open hours**	**1**
Week day working hours only	2
Week days and nights	0
Also opens at weekend	1

Some of experts buttressed some of the choices they made in rating the attributes and levels for importance and appropriateness with comments. Concerning the level *‘Other health worker’* of attribute *‘Preferred health worker’,* some reviewers were of the opinion that other cadres of health workers may not have relevant skills at the moment to provide ASRH services. However, they did argue that these persons remain a crucial source of the work force that supports ASRH services. One of the experts said: *“Other health workers such as Community health extension worker (CHEW) are also very important as they are community based, understand that these young people, willing to go extra miles with them and knows the community very well so must be well groomed not to judge, not to report to parents and ensure warm welcome to these young people in the facility”.* For the levels of the attribute *‘AYP Outreach/Awareness creation’*, one of the experts felt that it was important to offer SRH services to AYP on as many platforms as possible, hence, the need to take the services beyond the health facility. A comment given in this regard is as follows: *“…young people are not homogeneous, different things means differently to different categories of young people. some will never like to be seen at the facilities while some would. By providing at the facilities alone will definitely cut out large number of young people from receiving services of course”*. With respect to the attribute ‘Cost of services’, some experts considered ₦1500 (≈ USD3.61) too high with comments like *“Definitely too high a cost for young people to pay’.* We however retained that level to allow for more robust willingness to pay estimation*.* Other reviewers felt appropriate the cost of service would be relative to the type of service. One such reviewer said, *“The appropriate price to me, is relative. Even the young people spend and invest personal money in so many other things such as, clothes, hair do, cosmetic, junks, gadgets etc. if* [the service] *is made affordable they'll access it.”*

The experts were divided on the utility of a separate spaces for ASRH in public health facilities. This is reflected in the comments of one of the experts: *“This also has its advantages and disadvantages. The pros are that no one would really know what the youth has come for and so can access the services without any 'suspicion' but the cons are that youths may be intimidated with the fact that adults are present and so may not be very comfortable or come at all”*. The attribute-level *‘60 minutes’* was also considered a long waiting time before receiving treatment/attention by most standards. One reviewer commented further, “*The longer the wait, the more discouraging it will be for the youths”.* For the attribute *‘Opening hours’* one reviewer felt the levels as presented were additive rather than distinct. The comment given is as follows: “*The* [current] *construction of the attribute-levels is additive.* [As] *these will be presented in separate *[choice tasks]*, the specific hours should be described for each level, not as an addition to another level”.* The cue was to make each attribute-level distinct and standalone.

For a number of attributes, their levels as presented to the experts were the positive and negative extremes. Some of the experts rightly pointed out that this situation will lead to dominant alternatives. One expert said about the levels of attribute ‘Physical environment’, *“I do not think using opposites for the levels is appropriate. The second level will not appeal to any one normally. I suggest adding some other desirable attribute to the environment to see which one the adolescents prefer”.* We therefore adjusted the levels by using different levels of cleanliness. Similar to this is the attribute ‘*Health worker attitude’* in which one of the reviewers wrote: “*I suggest that some desirable attitudes should be added not the opposites. You are trying to see which one matters most to the adolescent”.* For the attribute*, ‘*Contraceptives’ availability’ a reviewer recommended the level ‘Always available’ be modified to read as ‘At least one method is always available’.

### Validation FGD with AYP

A total of 33 AYP participated in the final validation FGDs with AYP. Among these, 14 (42.4%) were 15 – 19 years old, 17 (51.5%) were female, 24 (72.7%) were Christians, 27 (81.8%) had completed senior secondary school, all were unmarried and of Yoruba ethnicity. This second round of FGD was an opportunity to review the specific attributes and levels developed as against the first round that was used to generate the attributes and levels. Each attribute was explored as *a priori* themes for the purpose of analysis. The subthemes that emerged from the discussion sessions were similar to earlier finding, with some new insight. The subthemes are shown in Table 3.

**Table 3:** Themes and subthemes emerging from the final FGDs

**Adolescent community outreach** • Needed • Not needed • Social media • Take to schools	**Contraceptive’s availability** • Available • Condom request made by man • For married ones • Free • Not for youths • Privacy in distribution • Should not be provided	**Physical environment/Amenities** • Adequate equipment • Attractive • Quietness • Conducive • Cleanliness • Clean toilets • Good equipment • Power supply • Water • Security • Beds with bed bugs
**Cost of services** • Low charges • Needs to be high enough • Subsidized • Willing to pay ₦1000 • Willing to pay ₦1500 • Willing to pay ₦2000 • Willing to pay ₦3000 • Willing to pay ₦3500 • Willing to pay ₦5000		
	**Health Worker attitude** • Caring • Good communication • Good relationship • Humble • Criticize • Nonchalant • Rude • Stigmatization	
		**Type of staff** • Doctor • Female to female • Nurse/Matron • Pharmacy • Very educated
**Open hours** • 6am - 6pm • 6am - 7pm • 7am - 7pm • 8am - 10pm • 9am - 4pm • Every time (24/7) • week days preferred • weekend preferred		
	**Waiting time** • Prompt attention • >30 minutes. • 30 minutes. • 60 minutes. • 2 hours • Booking in advance	
		**Privacy/Confidentiality** • Don't keep secrets • Given in the hospital • Private facility preferred • Separate place for Adolescents • Should keep secrets • Social media confidential

For *‘AYP Outreach/Awareness creation’*, the option of social media outreach was suggested. However, we did not consider this as an appropriate option for the public health facilities because of likely limited reach among the study population. Many participants felt that contraceptives should not be offered to AYP (at least the unmarried). There was general negative predisposition to providing them in the health facilities. While the participants suggested relatively high amounts as affordable by AYP for ‘cost of services’, it seemed more reasonable to only include relatively lower costs to avoid the problem of dominant alternatives. Similarly, numerous open hours were suggested which largely overlapped and needed to be harmonized to express them as distinct levels. For attitudes of health workers, the participants expressed these in either positive or negative terms in keeping with the original draft of the DCE tool.

Some participants suggested waiting times longer than one hour in some of the discussions but we considered it most useful not to extend waiting times beyond one hour to avoid ending up with alternatives having too extreme levels. The type of staff suggested generally mirrored the existing levels we had designed although some participants suggested the possibility of gender sensitivity in which female health workers attend to female AYP. Similarly, the suggestions under *‘Privacy and confidentiality’* mirrored the existing levels we had. Finally, when the wordings of each the existing attributes and levels were read out during each session, the participants generally confirmed that they understood the wordings of the attributes and levels. With respect to the ranking of the attributes, the ranking of the mean of ranks assigned by individuals and groups had a similar pattern in which the attributes *‘AYP Outreach/Awareness creation’*, *‘Privacy/Confidentiality’*, and *‘Contraceptives’ availability’* ranked the lowest (Table 4).

**Table 4:** Ranking of proposed attributes by mean of ranks assigned by FGD participant

**Attributes**	**Individual ranking**		**Group ranking**	
	**Mean of ranks (MOR)**	**Rank of MOR**	**Mean of ranks (MOR)**	**Rank of MOR**
Type of staff	3.38	1	2.50	1
Physical environment/Amenities	4.38	2	5.00	4
Cost of services	4.66	3	2.50	2
Open hours	4.70	4	5.00	6
Health Worker attitude	4.89	5	4.50	3
Waiting time	5.09	6	5.00	5
Adolescent community Outreach/Awareness	5.25	7	6.00	7
Privacy/Confidentiality	5.88	8	7.00	8
Contraceptive availability	6.56	9	7.50	9

### Final draft of DCE tool

To create the final draft of our survey tool, we had concerns about the number of attributes that will be going into choice tasks to avoid cognitive overload. We thought six or seven were appropriate for the target population since the number of attributes affects model estimates, especially the error variance of the model [30]. We also considered the option of splitting the nine existing attributes into two groups using efficient designs, meaning that we could retain all the attributes. However, the tradeoff would be the requirement for a much larger sample size in the survey. We therefore decided to exclude ‘*Privacy/Confidentiality’* attribute and its levels, as presented, on the account of the general recommendation that SRH services should continue to be offered alongside services for the general population [31, 32]. We also removed attribute ‘*AYP Outreach/Awareness creation*’ in order to allow for a focus on services provided within the public health facility. We retained *‘Contraceptives’ availability’* because this was the only attribute that directly linked the tool to reproductive health services. Also, we changed the attribute *‘Physical environ/Amenities’* to *‘Physical environment’* and included three levels for it. We modified the wordings of the levels of *‘Contraceptives’ availability’.* We changed the attribute *‘Opening hours’ to* ‘*Open hours’* and modified the wording of its level as well. The final draft is presented in Table 5.

**Table 5:** Final list of attributes and their corresponding levels

**Service Attribute**	**Levels**
Type of staff	Nurse
	Doctor
	Other health worker
Physical environment	Very clean (5 star)
	Moderately clean (3 star)
	Not clean at all (1 star)
Health worker attitude	Service provider is open and friendly
	Service provider is stern and may be judgmental
Cost	Free
	₦500
	₦1500
	₦2500
Waiting time	No wait
	30 minutes
	60 minutes
Contraceptive availability	At least one method is always available
	May not always be available
Open hours	Weekdays only (8am – 4pm)
	Weekdays and after hours (8am – 8pm)
	Weekdays and weekends

## Discussion

In this study, we followed a three-step process to arrive at the final attributes and attribute-levels for a DCE study tool. These were qualitative exploration to develop initial attributes and levels, an expert validation of the attributes and attribute-levels developed, and a final validation by adolescents and young people who are the target of the DCE. While there was a general agreement of the expert reviews with the initial set of attributes and attribute-levels derived from the first set of FGD, the experts used their knowledge to fine-tune and streamline the items we developed. Also, the final validation with another set of AYP confirmed and provided some evidence for workability of the attributes and attribute-levels based on the ranking exercise conducted. This rigorous approach has ensured that the final set of attributes and attribute-levels are contextually adapted to the study population of interest.

Our set of attributes and attribute-levels differ slightly from a number of other researchers that have done similar work. According to Armstrong et.al., typical characteristics of adolescent friendly services include that they provide services at convenient opening times, have minimal waiting time, have flexible appointment systems, have a welcoming and clean environment, and include adolescent-friendly spaces [33]. Of these, our tool captured four. We did not consider the performance of ‘adolescent-friendly spaces “strong enough to justify making it an attribute as it was only mentioned by one participant, in the second set of FGDs. Also, appointment making did not feature as a theme in the FGD. The work of Michaels-Igbokwe et al. which was similar to ours focused on contraceptives services for adolescents, while ours took a broader view of SRH services for AYP [10]. However, in order to prevent our tool from being too broad or generic for all services in a health facility, we deliberately retained an attribute for contraceptives services for AYP.

In this study, we started with an exploration of the perspective of members of the target population on whom the DCE tool will be administered, as recommended in recent guidelines [17]. Some authors started the development process from extracting initial draft of attributes and attribute-levels from literature review [34]. There are pros and cons on both sides. Starting from a literature review may remove the need to expend resources on the initial qualitative study. However, if existing literature is not rich enough there are chances that important attributes and levels will be left out. On the other hand, starting with qualitative research among the study’s target population already grounds the research within their context and allows the elicitation of the attributes and attribute-levels to be as close as possible to the typical circumstances under which the tool will eventually be used. With the understanding that the questions asked during the initial FGD will determine the responses, we created an FGD guide that explored the core concepts of accessibility, acceptability, privacy and confidentiality, affordability, and equitableness SRH services for AYP. The argument that can be made perhaps is to use a combination of review of literature and qualitative research to elicit the first draft of attributes and attribute-levels. In our case however, we used findings from qualitative research, refined them with expert opinions, including those from previous studies, and further validated them with users’ perspectives. These three steps provided both practical and technical basis for our attributes and attribute-levels.

Expert validation step was very critical to our attributes and attribute-level development. It was necessary to select both method (DCE) and content (SRH for AYP) experts. For instance, one of the methods experts pointed out the very important need to make attributes levels distinct yet close to give allowance for respondent to actually trade-off. Extreme levels of attributes would either be very desirable or not desirable at all, leading to dominance of some alternatives, and defeating the purpose preference elicitation [30]. However, not all types of DCE attribute development will need expert validation. This is perhaps especially true when the target population for the tool, and invariable the ones with whom preliminary qualitative research would be done, can be considered as experts. For instance, in a field like transportation, the transport service users may be considered as both the users and the experts in making transport service choices.

The final validation step allowed us to clarify the meaning of the wordings of our attributes and levels and yet have some quantitative object measure for excluding attributes through ranking of attributes. Although privacy and confidentiality are very important parts of AYFHS, and indeed any other type of services, we excluded it from our final set of items because it had a comparatively high average mean rank score (hence lower rating). We were more confident about excluding it because the attribute-levels that emerged along with it in the development process was about having separate space for AYP in health facilities. Evidence from literature suggests that separate space models have proven difficult to sustain and scale due to staff and resource shortages, and low utilization of available specialized services, among many other constraints [31]. It may be that if other options were given like ‘Privacy and confidentiality provided’ vs. ‘Privacy and confidentiality not provided’, the attribute may have been retained in the validation process. Future research can explore other possible levels of the attribute ‘privacy and confidentiality’. Also, we retained the attribute-levels of health worker attitude as was used in the work of Michaels-Igbokwe et al. [10].

We made efforts to guarantee credibility of our methodology and findings through sample diversity and maximum variation among the study' target population, triangulation of data and careful selection of meaningful units, with quotations to exemplify our choices. Our use of a FGD guide that captured the elements essential to optimal SRH for AYP enhances the dependability of our findings. Also describing the full process of our data management including context, participants, data collection, and analysis enhances the transferability of our findings. From a methodological point of view, our use of a modified Delphi approach and assessment of IQR as measure of agreement is a simple and objective way of including consensus in the attribute development process, especially expert consensus which required objective measurement.

Many researchers have used only qualitative methods in their attributes development approach, with or without initially deriving candidate attributes and levels from a literature review [15, 34, 35]. Kløjgaard and colleagues extended their qualitative methodology to add a quantitative priority listing like we have done in this study [19]. What is common to most is the multiple iterations before arriving at a final set of attributes and levels. In this study, we have used qualitative methods in multiple iterations with an addition of a quantitative expert validation step. These layered processes and additional quantitative expert validation provided increased rigor for the attributes development.

According to Coast et.al., [15], appropriate attributes for a preference study should have four characteristics namely: selected attributes should include all that are important for an individual in making a decision; none should be close in meaning to the construct been investigated; no single attribute should be much more influential than others in making a decision; and attributes should be characteristics of the construct of interest and not intrinsic to the person making the decision. The strength of our study lies in the process we have taken to ensure the characteristics in the attributes and levels we developed. We sought to optimize the attributes included in our tools through the elicitation of the items using qualitative research with the target population and validation of developed attributes and attribute-levels with content and methods experts. The process of elicitation and validation of the attributes and attribute-levels has also helped us to see to it that the attributes and attributes-levels are characteristics of, but individually distinct from, our latent construct, sexual and reproductive health services for adolescents. We eliminated the likelihood of occurrence of levels of attributes that are likely to lead to deterministic choices, hence dominance, by rewording. This was especially true for those levels that were extremes of one another, in which individuals are always likely to go with the more positive/favorable extreme. Finally, we consider none of the attributes selected as intrinsic to the individual choice makers.

### Limitations

The processes involved in developing the attributes and levels are generally reductionist in which the researcher has to continue to make decisions on what to include or exclude. Excluding some items tended to make the tool more generic and less specific to our latent construct of interest. We therefore needed to make value-based decisions for inclusion and exclusion as seen in our third decision rule of *“expert and practical knowledge of implementation of SRH services”.* This is seen, for instance, in our decision to retain the attribute ‘contraceptive availability’ in order to keep the tool grounded in SRH and not be too generic. It means that a different set of researchers could make a different set of decisions with the same set of data.

## Conclusions

The final set of attributes we developed in this study covered those relating to the services provided, the health workers providing the services, and the AYP. Much like DCE itself, deciding on which attributes and levels to include also involves trading off among items. Having well spelt out decisions rules for including and excluding attributes and levels at every phase is very important for transparency and reproducibility of the development process. The process we took guided us in making reasonable trade-offs among competing attributes in a transparent manner. Finally, adopting both qualitative and quantitative methods ensured a rigorous process to produce a robust combination of attributes and levels.

## Data Availability

The datasets used and/or analyzed during the study are available from the corresponding author on reasonable request.
